# Inhibitors of HIV-1 Reverse Transcriptase—Associated Ribonuclease H Activity

**DOI:** 10.3390/biology1030521

**Published:** 2012-10-19

**Authors:** Tatiana Ilina, Krystal LaBarge, Stefan G. Sarafianos, Rieko Ishima, Michael A. Parniak

**Affiliations:** 1 Department of Microbiology and Molecular Genetics, University of Pittsburgh School of Medicine, 450 Technology Drive, S.414, Pittsburgh, PA 15219, USA; Email: tai4@pitt.edu (T.I.); kml77@pitt.edu (K.L.); 2 Department of Molecular Microbiology & Immunology, University of Missouri, Columbia, MO, USA; Email: ishima@pitt.edu; 3 Structural Biology, University of Pittsburgh School of Medicine, 450 Technology Drive, S.414, Pittsburgh, PA 15219, USA; Email: sarafianoss@missouri.edu

**Keywords:** HIV-1, reverse transcription, ribonuclease H (RNase H), ribonuclease H inhibitor (RNHI)

## Abstract

HIV-1 enzyme reverse transcriptase (RT) is a major target for antiviral drug development, with over half of current FDA-approved therapeutics against HIV infection targeting the DNA polymerase activity of this enzyme. HIV-1 RT is a multifunctional enzyme that has RNA and DNA dependent polymerase activity, along with ribonuclease H (RNase H) activity. The latter is responsible for degradation of the viral genomic RNA template during first strand DNA synthesis to allow completion of reverse transcription and the viral dsDNA. While the RNase H activity of RT has been shown to be essential for virus infectivity, all currently used drugs directed at RT inhibit the polymerase activity of the enzyme; none target RNase H. In the last decade, the increasing prevalence of HIV variants resistant to clinically used antiretrovirals has stimulated the search for inhibitors directed at stages of HIV replication different than those targeted by current drugs. HIV RNase H is one such novel target and, over the past few years, significant progress has been made in identifying and characterizing new RNase H inhibitor pharmacophores. In this review we focus mainly on the most potent low micromolar potency compounds, as these provide logical bases for further development. We also discuss why HIV RNase H has been a difficult target for antiretroviral drug development.

## 1. Introduction

The viral enzyme reverse transcriptase (RT) is essential for replication of the human immunodeficiency virus (HIV), the causative agent of acquired immunodeficiency syndrome (AIDS). HIV RT is multifunctional, with both RNA-dependent and DNA-dependent DNA polymerase activity, as well as ribonuclease H (RNase H) activity that degrades the RNA component of the RNA/DNA hybrids duplex intermediate formed during reverse transcription. All of these RT activities are essential for transformation of the viral single-strand genomic RNA into double-strand DNA that can then be integrated into the host cell genome. 

HIV RT differs significantly from cellular DNA polymerases and it has become a major target for antiviral drug discovery and development. In mid-2012 over half of the FDA-approved drugs or drug combinations for the treatment of AIDS/HIV comprise inhibitors of RT DNA polymerase activity. These inhibitors comprise two different classes, nucleoside/nucleotide RT inhibitors (N(t)RTIs) and nonnucleoside RT inhibitors (NNRTIs), differing in structure and mechanism of action [1]. N(t)RTIs are RT active site-directed nucleoside analogs that require metabolic activation (phosphorylation) for antiviral activity. Once activated, NRTI-triphosphates and NtRTI-diphosphates compete with cellular deoxynucleotides for binding to the RT polymerase active site. More importantly, N(t)RTIs lack a 3’-OH hydroxyl on the sugar analogue moiety of the drug, thus once incorporated by RT into the viral DNA, extension is prevented and further viral DNA synthesis is blocked. In contrast, NNRTIs comprise a diverse group of chemical structures that bind to an allosteric site on RT distinct from the polymerase active site, and do not require metabolic activation for antiviral activity. NNRTIs are noncompetitive with respect to deoxynucleotide substrates and are considered to inhibit RT-catalyzed DNA polymerization by inducing conformational changes that alter RT active site geometry. However, the rapid mutation rate of HIV has led to the development of resistance to each of the clinically used antiretrovirals as well as viral variants with multi-class drug resistance, potentially impacting on the continued efficacy of current drug regimens. Continued drug discovery and development is essential, especially drugs directed at as yet underexplored steps of HIV replication [1,2]. HIV RT-associated RNase H activity is one such target. Accordingly, HIV has received increased attention over the past decade. The development of robust high throughput screening assays has enabled evaluation of hundreds of thousands of compounds as potential RNase H inhibitors (RNHIs), resulting in the identification of numerous RNase H-specific inhibitors with diverse chemical structures. A number of crystal structures of RNHIs in complex with the isolated RNase H domain or with intact RT have recently been published, providing a strong structural basis for further inhibitor development and optimization. This review summarizes recent progress in the discovery and development of small molecule inhibitors targeting HIV RT RNase H activity. 

## 2. HIV-1 RT RNase H Structure and Activity

**Figure 1:**
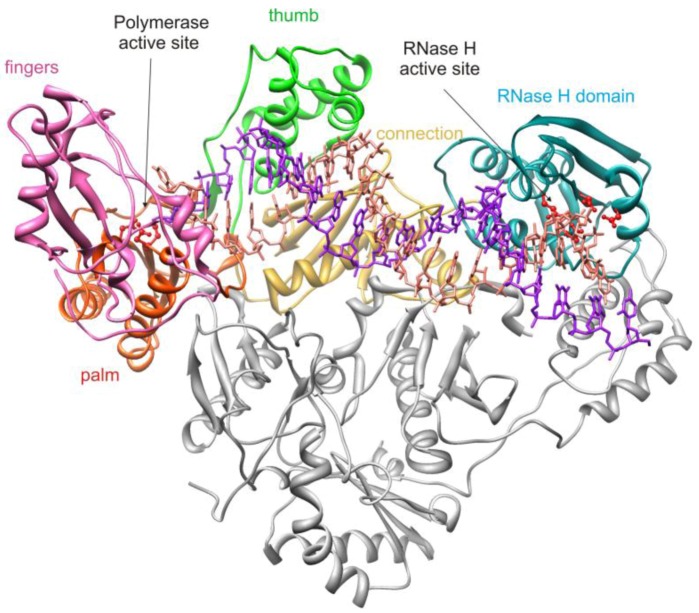
Structure of HIV-1 p66/p51 heterodimeric reverse transcriptase in complex with nucleic acid. The subdomains of the p66 subunit are depicted in different colors; the p51 subunit is depicted in gray. The red ball and stick residues in the RNase H domain denote the active site carboxylate amino acids. The figure is derived from PDB file 1RTD and was drawn using UCSF Chimera software [6].

HIV-1 RT is an asymmetric heterodimer consisting of 66 kDa (p66) and 51 kDa (p51) subunits with identical primary sequences with the exception of an additional 15 kDa C-terminal subdomain on the p66 subunit which comprises the RNase H domain of RT (Figure 1). All RT enzymatic activity is associated with the p66 subunit which contains both the polymerase and RNase H active sites, separated by approximately 40Å, a distance corresponding to 17–18 base pairs of an RNA/DNA duplex [3,4,5]. The polymerase active site is located within the palm subdomain with catalytic aspartic acid residues D110, D185 and D186. The p51 subunit is catalytically inactive and serves as a structural scaffold for the p66 subunit. The connection domain of p66 links the polymerase and RNase H domains and is critical for RT-nucleic acid interaction [5]. 

### 2.1. HIV-1 RT RNase H Catalytic Mechanism

**Figure 2:**
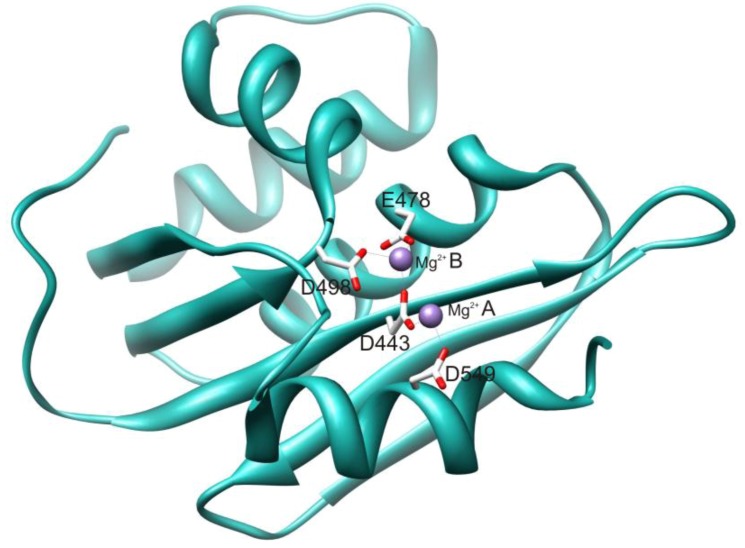
Structure of the RNase H domain of HIV-1 RT. Active site catalytic residues are depicted in stick form and the active site Mg^2+^cations are shown as spheres. The figure is derived from PDB file 3K2P and was drawn using UCSF Chimera software [6].

The HIV RT RNase H domain tertiary structure (Figure 2) is similar to all known RNase H enzymes, including human RNase H1, despite significant differences in primary sequence. The HIV RT RNase H active site contains four highly conserved catalytic acidic residues (D443, E478, D498 and D549) located in a cavity that also includes the essential H539 [7]. The catalytic DEDD motif coordinates with two Mg^2+^ cations that are essential for enzyme function. The RNase H primer grip is adjacent to the active site and interacts with the DNA strand of the RNA/DNA hybrid duplex nucleic acid substrate [4]. This interaction is critical for the proper binding and positioning of the hybrid duplex substrate in the RNase H active site, and impacts both on RNase H catalysis and on DNA polymerization [8,9,10]. Mutations of certain primer grip residues seriously abrogate RNase H activity [11,12].

**Figure 3:**
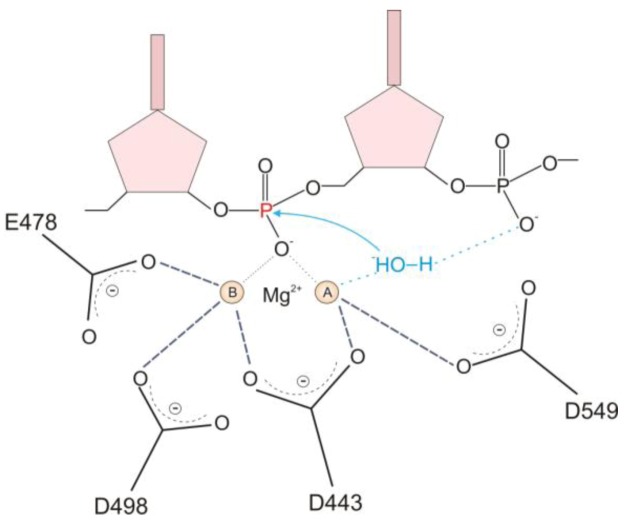
Schematic of the two-metal mechanism of RNase H hydrolysis. The active site metals are indicated as A and B. details are provided in the text. The figure was adapted from [14].

The mechanism of RNase H catalyzed hydrolysis involves a two-metal cation cleavage event [3,7]. Briefly, deprotonation of bound water by metal cation A results in formation of a hydroxyl ion (OH^−^) that attacks the 5’-scissile phosphate of the RNA strand leading to cleavage of the phosphodiester bond (Figure 3). Metal cation B interacts with the leaving group from hydrolysis to lower the activation energy of the transition state. Both metal cations are coordinated to and positioned in the active site by the catalytic residue tetrad D443, E478, D498 and D549 [13]. 

### 2.2. RNase H Hydrolytic Activity during Reverse Transcription

**Figure 4:**
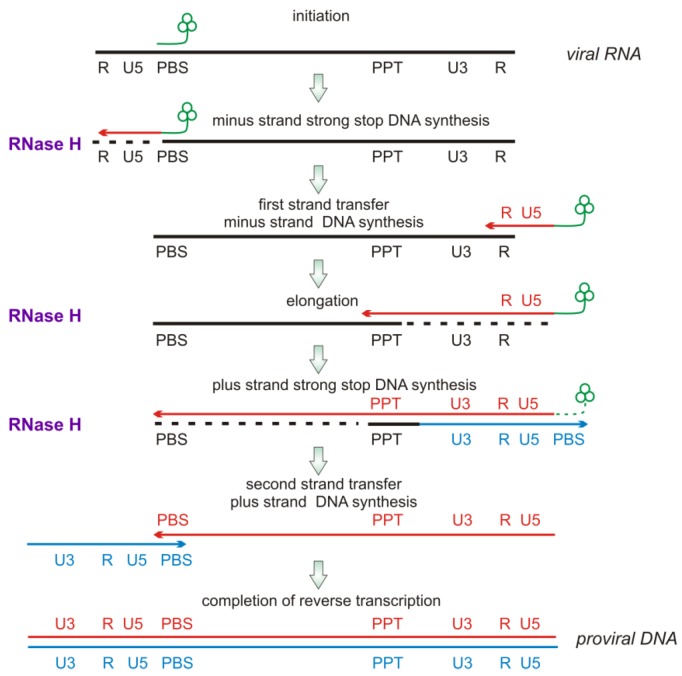
Schematic of the stages of reverse transcription. Stages where RT-RNase H functions are indicated by “RNase H” and a dashed line denoting RNA hydrolysis. Details are in the text.

HIV genomic information is in the form of (+) RNA, but HIV replication involves an obligatory conversion of this RNA into dsDNA that is incorporated into the infected host cell genome. HIV thus encodes for a specific enzyme, reverse transcriptase (RT) to carry out this process. Reverse transcription (Figure 4) initiates from an RNA primer provided by a specific cellular tRNA (tRNA^Lys3^) incorporated during virion assembly. The eighteen 3’-terminal nucleotides of this tRNA are annealed to a complementary sequence near the 5’ end of the HIV genomic RNA termed the primer binding sequence (PBS). RT-catalyzed RNA-dependent DNA synthesis then proceeds until RT reaches the 5´ end of the RNA genome, providing a strand of HIV (-) DNA complementary to the U5 and R terminal repeats of HIV genomic RNA. These newly synthesized sequences are essential for hybridization to the 3’-end of the HIV genomic RNA template to enable completion of full length (-) DNA synthesis. However, the (-) DNA sequences are in the form of an RNA/DNA hybrid duplex. The RNA strand of this duplex must be removed to allow hybridization of the newly synthesized viral (-) DNA with the terminal repeat region of the 3’-end of the viral RNA. The RNase H activity of RT removes this RNA strand, enabling strand transfer and continuation of reverse transcription. If the RNA strand is not removed, reverse transcription and HIV replication stop [14]. After the first strand transfer, RT DNA polymerase activity continues (-) DNA synthesis and RT-associated RNase H degrades the template RNA. During this process a purine-rich sequence of HIV genomic RNA, the polypurine tract (PPT), is generated. The PPT in duplex with complementary DNA is somewhat refractory to RNase H-catalyzed degradation, and serves as a primer for synthesis of the HIV (+) DNA strand [5]. RT RNase H removes the PPT RNA component after priming of (+) DNA synthesis. Following sufficient elongation, the PPT RNA component is degraded, again by RNase H. Viral (+) DNA synthesis continues including that part of the tRNA initiation primer still associated with the (-) DNA. RT-RNase H activity then acts to remove the tRNA component still associated with the nascent viral DNA. RT RNase H activity is thus essential at several stages of HIV replication. 

### 2.3. Modes of RNase H Hydrolysis

**Figure 5:**
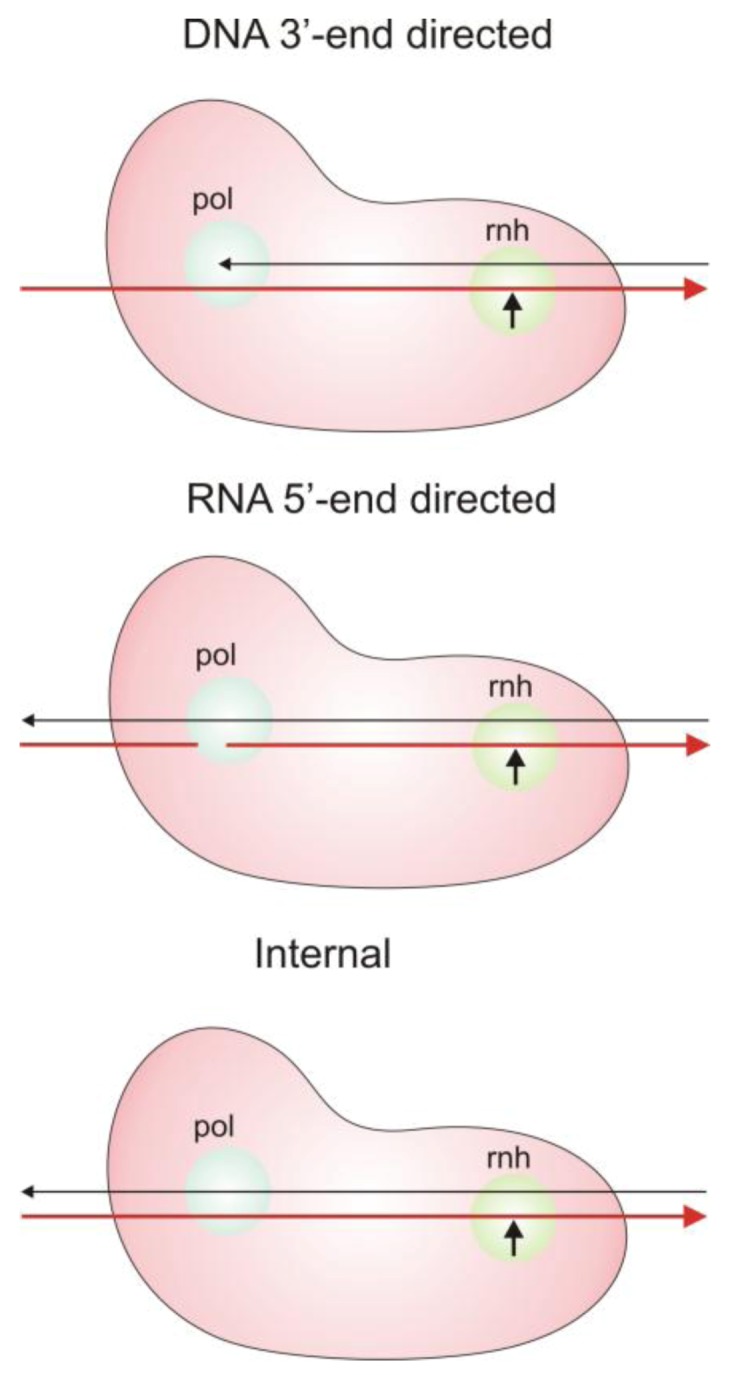
Modes of RT RNase H cleavages. The DNA strand of the nucleic acid duplex is black and the RNA strand is red. Details are provided in the text. Figure adapted from [14].

The critical requirement for RT RNase H activity at multiple stages of reverse transcription necessitates at least three distinct modes of RNase H cleavages, based on the mode of interaction of the RNA/DNA hybrid duplex substrate with RT (Figure 5).

*3’-DNA directed or polymerase-dependent cleavages*. During active DNA polymerization, the 3’-end of the growing DNA strand is positioned in the RT polymerase active site; this orients the RNA template in the RNase H active site such that cleavage occurs 17–18 nucleotides downstream from the ribonucleotide complementary to the primer 3’-terminus [13,14]. This suggests that if RT polymerase and RNase H activities function in a concerted manner, the downstream RNA template will be degraded as the new DNA strand progresses. However, the rate of RT-catalyzed nucleotide incorporation is in fact much greater than that of RT-associated RNase H hydrolysis [15]. Thus, during processive RT-catalyzed DNA synthesis, 3’-DNA directed RNase H cuts likely occur only when polymerization pauses due to secondary structural features such as hairpins in the viral genomic RNA template. Substantial stretches of RNA remain uncleaved and duplexed to the growing DNA strand, interspersed with ‘nicks’ arising from RNase H cuts due to polymerization pausing. Removal of these large segments of residual RNA is carried out by two different polymerase independent cleavage modes. 

*5’-RNA directed or polymerase-independent cleavages*. In this cleavage mode a recessed 5’-end of the RNA template strand positions the DNA strand in the polymerase active site such that the RNase H domain localizes to carry out cleavages 13-17 nucleotides downstream of the 5’-RNA terminus [13,14]. The exact cleavage position may depend in part on the sequence of the RNA strand [14,16,17]. 

*Non-directed or internal cleavages*. In this mode, cleavages take place within large segments of RNA/DNA duplex, and are not dependent on any positioning of the nucleic acid termini within the RT polymerase site, but are dependent in part on the sequence of the RNA [13,15,16]. These internal cleavages are abundant during reverse transcription. 

Infectious HIV virions contain two copies of the genomic RNA template, thus it is possible that DNA polymerase activity requires only one or two RT molecules. However virions contain multiple copies of RT, and it is probable that most, if not all, of the excess RT molecules are involved in RNase H cleavage. Indeed, recent data from our laboratory suggests that even modest reductions in HIV RNase H activity result in significant attenuation of virus replication [18]. As described above, the polymerizing RT generates nicks in the RNA during polymerization pausing events, but these would occur too infrequently to allow facile dissociation of the RNA strand from the newly synthesized DNA. Additional nicks are generated by RNase H internal cleavages carried out by non-polymerizing RT molecules. When the nicks are close enough (8–12 nucleotides depending on sequence), that small segment of RNA could dissociate from the DNA strand, providing a recessed 5’-RNA terminus that would provide a substrate for 5’-RNA directed RNase H cleavages, also carried out by non-polymerizing RT molecules. Continued interplay among the three different types of RNase H cleavage eventually degrades the RNA strand sufficiently to free up the DNA to serve as template for second strand DNA synthesis and completion of reverse transcription. Each of the different binding modes for interaction of RT RNase H with the RNA/DNA duplex likely represents a distinct macromolecular complex or mechanistic form of the enzyme and it is possible that the relative rates of cleavage of the RNA strand differs in each of these different complexes. We previously showed that NNRTIs have differential inhibitory potency against different mechanistic forms of RT polymerase [19], and it is probable that RNase H inhibitors (RNHIs) may also differentially inhibit the different mechanistic forms of RNase H. This possibility has not been explored in RNHI discovery programs. 

## 3. Inhibitors of HIV-1 RT RNase H

RT RNase H is essential for HIV replication, playing critical roles at several stages of reverse transcription. Furthermore, none of the major mutations associated with HIV resistance to clinically used antiretroviral drugs are found in the RT RNase H domain. RNHIs that specifically bind in or near the RT RNase H domain would therefore likely retain potency against clinically significant drug-resistant HIV variants, including multidrug resistant viruses. Yet less than a decade ago, only a handful of small molecule “drug-like” RNHIs had been described [1], due in large part to the time-consuming assay methodologies needed to assess RNase H activity. Two factors contributed to the recent increased pace of RNHI discovery. First was the development of raltegravir, a therapeutic HIV integrase inhibitor drug that works in large part due to interaction with the divalent metal cations in the integrase active site [20,21]. RT RNase H has both essential active site divalent metal cations and structural similarity with HIV integrase [22], providing a logical focus on integrase-inhibitor chemotypes. In the same context however, structural similarity with human RNase H1 raises concerns for potential off-target activity. Second was our development of a robust fluorescence-based assay, adaptable to robotic high throughput screening [23,24,25]. As of mid-2012, numerous small molecule RNHIs have been published. By analogy to RT polymerase inhibitors, RNHIs likely classify as active site inhibitors or allosteric inhibitors. Although most RNHIs have not been adequately studied for mechanism of action, this is reasonably suggested by their structure. Several previous reviews have provided excellent overviews of RNHI discovery and development up to approximately 2010 [26,27,28]. In the present review, we focus primarily on newly identified inhibitors as well as on those classes of inhibitor with potent activity (low to sub-micromolar potency *in vitro*), relative specificity for RNase H and with the potential for further optimization. We also include compounds for which structures of the inhibitor-RNase H complex have been obtained, as these provide a basis for future structure-based drug design. 

### 3.1. Active Site-directed RNase H Inhibitors (Table 1)

The design of RNase H active site-directed inhibitors has been the major focus in the pharma effort to develop potential RNHI therapeutics. To date, all active site-directed RNHIs are based on pharmacophore structures with strategically positioned functionality to enable interaction with the two metal cations in the RNase H active site. This interaction is expected to block access of the metals to the scissile phosphodiester bond in the RNA strand of the bound nucleic acid substrate, thereby preventing the metal-catalyzed hydrolysis reaction (Figure 3). 

The **diketo acid** (DKA) pharmacophore (Table 1, structure *1*) arose from the Merck integrase (IN) inhibitor development program [20,21]. Due to the presence of active site metal cations and the structural similarities between HIV IN and the RT RNase H domain, DKAs initially developed as integrase inhibitors were evaluated for potential inhibition of HIV-1 RNase H activity [19]. Among the most potent inhibitors was 4-[5-(Benzoylamino)thien-2-yl]-2,4-dioxobutanoic acid (BTDBA) [22] (Table 1, structure *1a*). Inhibition of RNase H by this compound was dependent on the presence of metal cations, and BTDBA inhibited a catalytically active RT RNase H domain fragment, binding to the protein with a 1:1 stoichiometry. It is thus probable that BTDBA binds within the RNase H active site, directly interacting with active site metal ions. This possibility is reinforced by the observation that BTDBA also has moderately potent inhibitory potency against HIV IN [22]. However, BTDBA showed no inhibitory activity against cell-based HIV replication. Tramontano *et al* reported that the DKA 6-[1-(4-fluorophenyl)methyl-1H-pyrrol-2-yl)]-2,4-dioxo-5-hexenoic acid ethyl ester (RDS-1643) showed relatively weak but selective inhibitory activity against RT RNase H (IC_50_ ~ 13 µM) and was able to inhibit HIV replication with similar potency [29]. However, HIV RNase H has not yet been validated as the target in this antiviral activity. 

**Figure 6:**
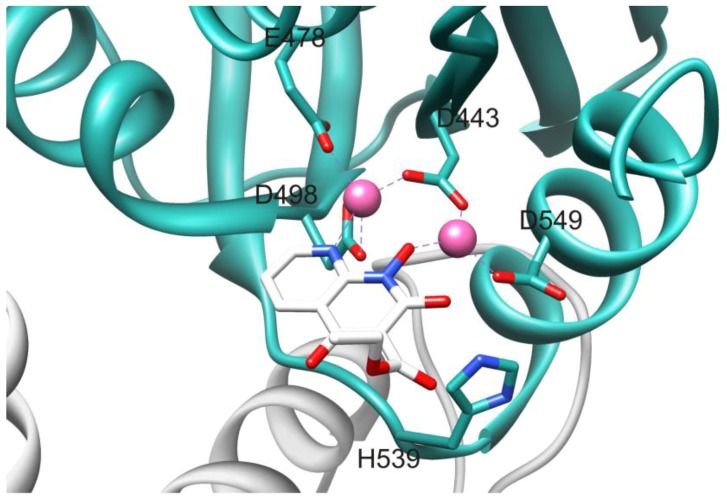
Binding of the N-hydroxy naphthyridinone inhibitor MK1 to the RT RNase H active site. The figure is derived from PDB file 3LP0 and was drawn using UCSF Chimera software [6]. Dashed lines indicate interactions with the metal cations.

The **N-hydroxy naphthyridinone** RNHI scaffold (Table 1, structure *2*) also derives from the Merck integrase inhibitor program [30]. The lead RNHI in this series, MK1 (Table 1, structure *2a*) inhibited RT RNase H *in vitro* with sub-micromolar potency but did not inhibit RT DNA polymerase activity [31]. While MK1 showed good antiviral activity (EC_50_ = 2.5 μM), this antiviral effect cannot be attributed to inhibition of RNase H since MK1 also inhibited integrase *in vitro* with sub-micromolar potency. Crystal structures of MK1 in complex with intact RT showed the inhibitor binding in the RNase H active site primarily by interaction with the two catalytic metal cations but also by possible interactions of the 3-substituent with H539 and N474 of the RNase H domain (Figure 6). 

**Table 1:** Active site inhibitors of HIV RT RNase H.

Pharmacophore	Example	IC_50_ (µM)				Reference
		RNase H	RT pol	IN	HIV	
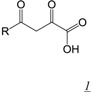	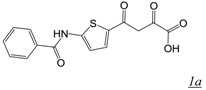	3.2	Not active	1.9	Not active	[22]
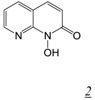	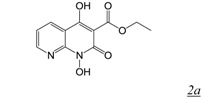	0.11	Not active	No report	2.8	[31]
	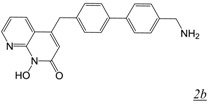	0.045	13	24	0.2	[32]
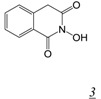	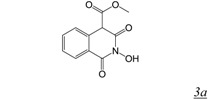	0.06	> 50	4.9	13.4	[36]
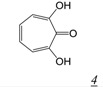	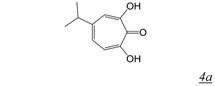	0.2	Not active	No report	Not active	[37]
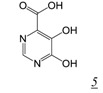	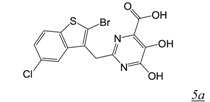	0.17	No report	No report	No report	[39]
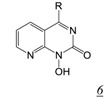	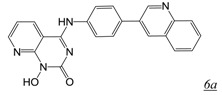	0.003	No report	0.4	0.01	[41,42]

A series of 4-substituted N-hydroxy naphthyridinones with lipophilic biaryl substitutions at the 4-position were prepared in order to take advantage of these potential additional contacts within the RNase H active site [32]. The approach was modestly successful with the most potent compound in this series (Table 1, structure *2b*) showing about a 2-fold increased RNase H inhibitory potency *in vitro* compared to MK1. Strikingly, the reported antiviral activity of this 4-substituted analogue was sub-micromolar (EC_50_ < 0.2 μM) [32]. Unfortunately, we have been unable to reproduce these data in cell-based HIV replication studies as in our hands the compound is cytotoxic so that the *in vitro* specificity of the inhibitor is insufficient to enable estimation of antiviral activity. 

The **N-hydroxyimide** RNHI pharmacophore was based on inhibitors of influenza virus endonuclease designed by a group at Roche to interact with a two metal-ion active site [33,34]. The basic pharmacophore, 2-hydroxy-(4H)-isoquinoline-1,3-dione (Table 1, structure *3*) specifically inhibited both intact RT-RNase H and a catalytically active RT RNase H domain fragment *in vitro* with sub-micromolar potency, but was inactive against RT polymerase activity as well as *E. coli* RNase H [25]. The position and angles of the three oxygens in the N-hydroximide moiety are such that they mimic the enzyme active site metal ion interaction with the substrate during catalysis and thus would be expected to be competitive inhibitors of RNase H catalysis. Crystal structures of the isolated RT RNase H domain in complex with N-hydroxyimide inhibitors confirmed that the compounds bind primarily by interacting with RNase H active site metals [33,34]. Unfortunately, none of the compounds was able to inhibit cell-based HIV replication. The same pharmacophore figures in a series of 7-substituted 2-hydroxyisoquinoline-1, 3(2H, 4H)-diones designed to be dual inhibitors of both HIV RNase H and integrase (IN) [35]. All of the initial series of 17 derivatives were substantially more potent inhibitors of integrase than RNase H, and none showed antiviral activity in the absence of cytotoxicity. SAR studies showed that all three oxygen atoms are essential for RNase H inhibition [36]. Continued development of the N-hydroxyimide pharmacophore has resulted in 2-hydroxy-4-methoxycarbonylisoquinoline-1, 3(2H, 4H)-dione [36] (Table 1, structure *3a*). This compound inhibits RT RNase H *in vitro* with nM potency (IC_50_ = 61 nM). It also inhibits HIV integrase but with two orders of magnitude less potency (IC_50_ ~ 5 µM). While this compound shows weak antiviral activity (EC_50_ ~ 13 µM), it is likely this is due primarily to inhibition of IN rather than RNase H.

The **tropolone **RNHI pharmacophore (Table 1, structure *4*) was identified from screening a library of natural products [37]. The most potent inhibitor, β-thujaplicinol (Table 1, structure *4a*) showed sub-micromolar inhibitory activity against both HIV-1 and HIV-2 RT RNase H, but much reduced potency against human RNase H and *E. coli* RNase H. The tropolones did not inhibit RT DNA polymerase activity. The geometry of the three oxygens on the 7-membered tropolone ring suggested that these might interact with the two metal cations in the RNase H active site, confirmed by crystal structures of β-thujaplicinol in complex with RT and an isolated RT-RNase H domain fragment [38]. Unfortunately, none of the tropolone RNHIs shows antiviral activity.

Kirschberg *et al* at Gilead designed the **pyrimidinol carboxylic acid **(PCA) (Table 1, pharmacophore structure *5*) RNHI pharmacophores from structural analysis of three other previously reported metal chelating RNHIs (DKAs, N-hydroxyimides and tropolones) [39,40]. The metal chelating functionality of pyrimidinol carboxylic acids is similar to that of the DKA class, but PCAs provide a more stable tautomeric scaffold than the DKA pharmacophore. Aryl substituents were introduced at C2 to provide additional protein contacts with H539, similar to the approach used for the 4-substituted N-hydroxy naphthyridinones (Table 1, structure *5a*). Crystal studies of these inhibitors in complex with the isolated RNase H domain of HIV RT showed that these compounds bind in the RNase H active site with primary interactions with RNase H active site metals as well as with H539 [39]. However, none of these compounds were reported to have antiviral activity.

Structure-based drug design is a major focus in drug discovery and solution of the crystal structures of several different active site-directed RNHI pharmacophore classes in complex with HIV RNase H should provide an excellent basis for RNHI optimization. However, the focus on metal interaction is not sufficient to provide potent inhibitors as the binding affinity this metal interaction imparts to small molecule chelators is unlikely sufficient to compete with the large RNA/DNA duplex which has multiple binding interactions with RT both within and outside the RNase H active site. The addition of substituents on the metal binding core to enable additional protein interactions (H539 and other RNase H residues) as done for the N-hydroxy naphthyridinones and the PAC inhibitors results in increased binding affinity, but still insufficient to adequately compete with the nucleic acid substrate encountered during reverse transcription. Indeed, this inability of the RNHIs to compete with the nucleic acid during HIV replication may account in part for the lack of antiviral activity with current active site-directed compounds. However, there is a recent potential breakthrough in this area. At the 2012 Cold Spring Harbor Retroviruses conference, Gerondelis reported on the development of pyrido-pyrimidinone compounds (Table 1, structure *6*) that inhibit both RT RNase H and HIV replication with low nM potency [41]. Several hundred analogues of this inhibitor class have been synthesized [42], some of which, such as GSK5724 (Table 1, structure *6a*), have exceptional RNase H inhibitory potency and antiviral activity. While this compound also inhibits IN, this inhibition is two orders of magnitude weaker than that for inhibition of RNase H and substantially less than the antiviral potency. It is exciting to speculate that the antiviral activity of GSK5724 arises from inhibition of RT RNase H during intracellular HIV reverse transcription.

### 3.2. Allosteric RNase H Inhibitors (Table 2)

Allosteric inhibitors of HIV RT DNA polymerase activity (NNRTIs) have proven therapeutic utility [1]. Allosteric inhibitors of RT RNase H would not directly bind in the active site and thus would less likely be displaced or competed out by the higher affinity nucleic acid substrate. Computational studies have identified potential allosteric binding pockets for identified RNHIs [43]. However, this class of RNHI has not received the same discovery and development effort as active site-directed RNHIs, and to date only a few compounds have been identified as probable allosteric RNHIs. There is considerable evidence that binding of NNRTIs as well as mutations in the allosteric pocket in the RT DNA polymerase domain impact on the activity of the spatially remote RT RNase H [44,45,46]. The mechanisms involved in this long-range alteration of RNase H activity are not entirely clear but likely involve changes in the positioning of the RNA/DNA duplex nucleic acid due to protein conformation changes in the polymerase domain following NNRTI binding. However, the effect of NNRTIs on RT RNase H activity is much less than on RT DNA polymerase activity.

**Thiocarbamates** and **1,2,4-triazoles **(Table 2, pharmacophore stuctures *7* & *8*) were identified as inhibitors of HIV RT RNase H through an HTS initiative at Wyeth [47]. The most potent inhibitor in each class is shown in Table 2, structures *7a* and *8a* respectively. Many of the identified inhibitors showed antiviral activity although the extent to which this was mediated by inhibition of RNase H is unclear as the compounds also inhibited RT DNA polymerase. Interestingly, both computational studies and crystallography show that triazoles bind in the NNRTI binding pocket in the RT DNA polymerase domain [48,49,50]. There are no structural data for interaction of triazole inhibitors with the RT RNase H domain. 

We have also identified a number of triazole RNHIs similar to those described in [47]; our most active inhibitor is structure *8b* (Table 2) that also has excellent antiviral activity. Interestingly, this compound does not inhibit a catalytically active isolated RT RNase H domain fragment. Furthermore, mutations in the NNRTI binding pocket associated with resistance to NNRTIs result in significantly reduced triazole inhibition of RT RNase H *in vitro* as well as a loss of antiviral activity in cell-based HIV replication assays [48]. These observations suggest that triazole RNHIs exert their inhibitory activity through binding to the RT polymerase NNRTI binding site. RNHIs that exert their effects *via* interaction with this site are not ideal as they would antagonize NNRTI binding and thus antagonize an entire class of clinically beneficial therapeutics. Furthermore, resistance to these RNHIs would certainly involve mutations in the NNRTI binding pocket which would likely confer cross-resistance to the NNRTI class of drugs. Nonetheless, structural and mechanistic information of how these NNRTI-site binding RNHIs exert their inhibitory activity may prove useful in the design of future novel NNRTIs with dual function inhibition (RT polymerase, RT RNH) *via* binding to a single site on the enzyme.

**Table 2:** Allosteric RNH inhibitors.

Pharmacophore	Example	IC_50_ (µM)			Reference
		RNase H	RT pol	HIV	
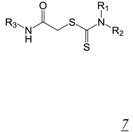	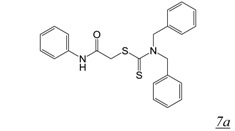	1.9	Not active	3.7	[47]
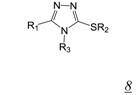	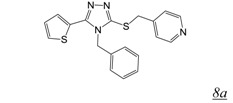	0.2	11.6	4.0	[47]
	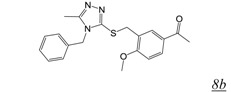	0.8	4.5	0.2	[48]
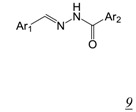	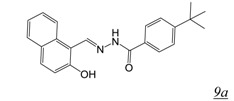	3.5	0.8	1.5	[51]
	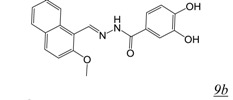	0.5	Not active	5.5	[52]
	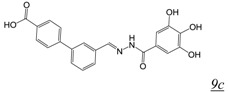	0.1	0.3	2.5	[54]
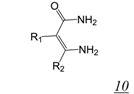	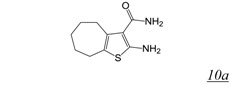	2.0	Not active	Not active	[55]

*in vitro*A number of **acylhydrazones** (Table 2, pharmacophore structure *9*) have been identified as RNHIs. We were the first group to describe a small molecule with low micromolar inhibitory activity against HIV RT RNase H, N-(4-tert-Butylbenzoyl)-2-hydroxy-1-naphthaldehyde hydrazone (BBNH) (Table 2, structure *9a*), a metal binding compound that also showed antiviral activity although with a narrow *in vitro* therapeutic window [51]. BBNH is in fact a dual function inhibitor, inhibiting both the RNase H and DNA polymerase activities of HIV RT. A variety of kinetic and biophysical measurements led to the suggestion that the dual function inhibition of BBNH might be due to interaction with two different sites on RT [51]. Early molecular modeling studies predicted that BBNH inhibition of RNase H might be due to binding in or near the active site *via* interaction with RNase H metal cations [11]. Inhibition of RT DNA polymerase was proposed to arise from binding to a site in the polymerase domain differing from that for NNRTIs. Further development resulted in additional antiviral analogues of BBNH with reduced metal binding and improved cytotoxicity, such as dihydroxybenzoyl naphthyl hydrazone (DHBNH) (Table 2, structure *9b*). Unlike BBNH, DHBNH inhibits only the RNase H activity of RT and is without effect on RT-catalyzed processive DNA synthesis [52]. A crystal structure at 3.15 Å resolution of DHBNH in complex with intact HIV RT showed the inhibitor to bind in the RT polymerase domain, near but not within the NNRTI allosteric binding pocket, but surprisingly no inhibitor was noted in the RNase H domain [52]. It was therefore proposed that binding of DHBNH to the polymerase domain could impact on RNase H activity by altering the trajectory of the nucleic acid due to observed structural changes in the polymerase primer grip, thereby preventing proper orientation of the RNA/DNA duplex substrate in the RNH active site. However, we consider it likely that DHBNH also binds in or near the RNase H domain of RT. The development of HIV resistance to DHBNH correlates with mutations (V254D, D256G, L260F) in the thumb subdomain of the RT p51 subunit, a region that contacts the RNase H domain in the RT p66 subunit (see Figure 1) [53]. We recently used protein NMR analysis to demonstrate interaction of the acylhydrazone BHMP07 (Table 2, structure *9c*) with an isolated RT RNase H domain fragment [54,55]. Superposition of the residues perturbed in the RNase H domain fragment onto the structure of intact RT suggests that BHMP07 binds to a pocket in the interface between the p51 subunit and the RNase H domain of the RT p66 subunit. Importantly, mutation of residues within this putative pocket leads to the loss of RNase H inhibitory activity of BHMP07 and of DHBNH [54]. Finally, recent computational studies have suggested that hydrazine RNHIs can readily dock to an allosteric pocket in the interface between the RT p51 subunit and the RT RNase H domain [43,55]. 

Screening of a library containing about 230,000 synthetic compounds as well as natural products for potential RNHIs identified the **vinylogous urea **pharmacophore (Table 2, structure *10*) [56,57,58]. Compound NSC727447 (Table 2, structure *10a*) was among the most potent, inhibiting HIV-1 RT RNase H with low micromolar potency *in vitro*. A combination of protein footprinting [56] and mutagenesis [58] approaches showed that vinylogous ureas interact with residues in the RT p51 thumb at the interface with the p66 RNase H domain, reminiscent of acylhydrazone interaction [43,53,54]. 

## 4. Conclusions

### 4.1. Unresolved Issues with RT RNase H as A Target for Antiretroviral Drug Discovery

The development of robust robotic HTS assays for inhibitors of HIV RT RNase H by us and by others has enabled a substantially increased pace for new inhibitor discovery, and as of mid-2012 numerous small molecule RNHIs with very good inhibitory potency against RNase H *in vitro* have been published. Unfortunately, very few of these show antiviral activity in cell-based HIV replication assays. Furthermore, there is no definitive evidence that any antiviral RNHI functions by inhibiting RT RNase H during HIV replication. Virtually all identified RNHIs with demonstrable antiviral activity, particularly the metal directed active site inhibitors, also inhibit other essential HIV activities such as integrase or RT DNA polymerase. RT RNase H has proven to be a very difficult target for antiretroviral drug development leading to a diminution of pharma interest in RT RNase H as a potential therapeutic target. 

Ideally, an inhibitor of a pathogen enzyme should target the rate-limiting step in that enzyme’s mechanism of action. Unfortunately, RT RNase H has received very little detailed mechanistic study as compared to RT DNA polymerase. As discussed in section 2.3, RT RNase H carries out a number of different types of RNA cleavages during reverse transcription. It is still unclear which of these is rate-limiting during reverse transcription. Identification of the rate-limiting process and development of HTS assays that specifically address this activity may assist in the discovery of RNHIs with therapeutic potential.

It has been suggested that therapeutic use of RNHIs may elicit resistance to NRTIs that are essential components in first-line treatment of HIV infection [59]. NRTIs lack a 3’-hydroxyl and thus act as terminators of RT-catalyzed DNA synthesis. A major mechanism of HIV resistance to NRTI therapeutics is the ability of RT to catalyze the phosphorolytic removal of the incorporated 3’-terminating NRTI [1]. According to this hypothesis, RNHIs would reduce the ability of the RNA/DNA duplex to translocate during RT-catalyzed processive DNA synthesis and thus increase the opportunity for phosphorolytic removal of the terminating inhibitor, thereby leading to apparent HIV resistance to NRTIs. Such potential antagonism is obviously unacceptable. The paucity of RNHIs with sufficiently potent antiviral activity has precluded direct testing of this hypothesis. It is also important to note that this antagonism, if it occurs, is likely to be expressed only by the actively polymerizing RT molecule, in other words, by the enzyme carrying out 3’-DNA directed RNase H cleavages. As discussed previously, 5’-RNA directed and internal cleavages likely represent the majority of RNase H cleavage events during HIV reverse transcription and these are catalyzed by RT molecules that are not actively polymerizing viral DNA. RNHIs specifically inhibiting these latter cleavages would not impact on HIV resistance to NRTIs. 

### 4.2. Future Efforts

Numerous small molecule RNHIs have been published since 2003. It is likely that many others have been identified but not yet publicly disclosed. Indeed, as yet unpublished screening efforts in our laboratory alone have already identified many new RNHIs of diverse chemotypes. We are presently developing a publically accessible RNHI database to provide our validated RNHI screening hits to the scientific community; we anticipate launch of this site mid-late 2013. We encourage others with screening data to submit information and those of any other groups who wish to contribute. 

Most screening efforts to date have used our HTS assay which employs a small 18 base-pair blunt ended RNA/DNA duplex designed to be highly sensitive to inhibition. We have now developed validated HTS screening substrates that enable screening for inhibitors of specific RNase H cleavages such as 5’-RNA directed cuts [60]. Use of these new substrates to re-evaluate our already identified inhibitors, as well as for screening of additional libraries for new inhibitors, may provide a better focus for identification of compounds with potential antiviral activity. Finally, the increasing numbers of structures of RNHIs in complex with the isolated RT RNase H domain and with intact RT provide an excellent basis for optimization of identified inhibitors and especially for future structure-based inhibitor design. 
